# Oxidized Low-Density Lipoprotein Induces WNT5A Signaling Activation in THP-1 Derived Macrophages and a Human Aortic Vascular Smooth Muscle Cell Line

**DOI:** 10.3389/fcvm.2020.567837

**Published:** 2020-11-19

**Authors:** Ian Ackers, Candice Szymanski, Mitchell J. Silver, Ramiro Malgor

**Affiliations:** ^1^Osteopathic Heritage Foundation, Translational Biomedical Sciences Program, Ohio University, Athens, OH, United States; ^2^Department of Biomedical Sciences, Ohio University, Athens, OH, United States; ^3^Midwest Cardiology Research Foundation, Columbus, OH, United States; ^4^The Diabetes Institute, Ohio University, Athens, OH, United States; ^5^Molecular and Cellular Biology Graduate Program, Ohio University, Athens, OH, United States

**Keywords:** macrophages, vascular smooth muscle cells, foam cells, Wnt signaling, vascular biology

## Abstract

The pathogenesis of atherosclerosis is complex, evolves, and involves many cell types. Macrophages and vascular smooth muscle cells (VSMCs) are critically involved in atherosclerosis development and progression. Several studies have shown that WNT5A protein is abundantly expressed in human atherosclerotic lesions; however, the mechanism and role of WNT signaling pathway activation is not clearly known. Using THP-1 derived macrophages, and human aortic VSMC cells, we evaluated *in vitro* how oxidized low-density lipoprotein (oxLDL) and WNT5A signaling interact in these two cell lines. We used western blot, scratch assay, metabolic proliferation assay, as well as immunostaining to analyze the effect of Wnt signaling activation. The results demonstrated that oxLDL, as well as WNT5A (control), induced Disheveled-2 (DVL2) activation and Kif26b degradation, indicating activation of non-canonical Wnt signaling. We found that oxLDL and WNT5A induced FZD5-ROR2 co-localization at the cellular membrane *in vitro* in THP-1 derived macrophages. Box5 (FZD5 receptor antagonist) inhibited oxLDL-induced DVL2/JNK activation secondary to newly secreted WNT protein from THP-1 derived macrophages. We found that WNT3A (canonical Wnt) and WNT5A showed different roles in this VSMC cell line. These findings indicate that WNT5A is upregulated by oxLDL, promotes foam cell formation, and affects VSMC phenotype and migration in these two cell lines. Also, in these cell lines FZD5 signaling seems to be necessary for lipid accumulation and, through this mechanism, WNT5A could modulate foam cell formation. Thus, our results suggest that WNT5A may contribute to the pathogenesis of vascular disease through modulating macrophage and VSMC behavior.

**Graphical Abstract:**
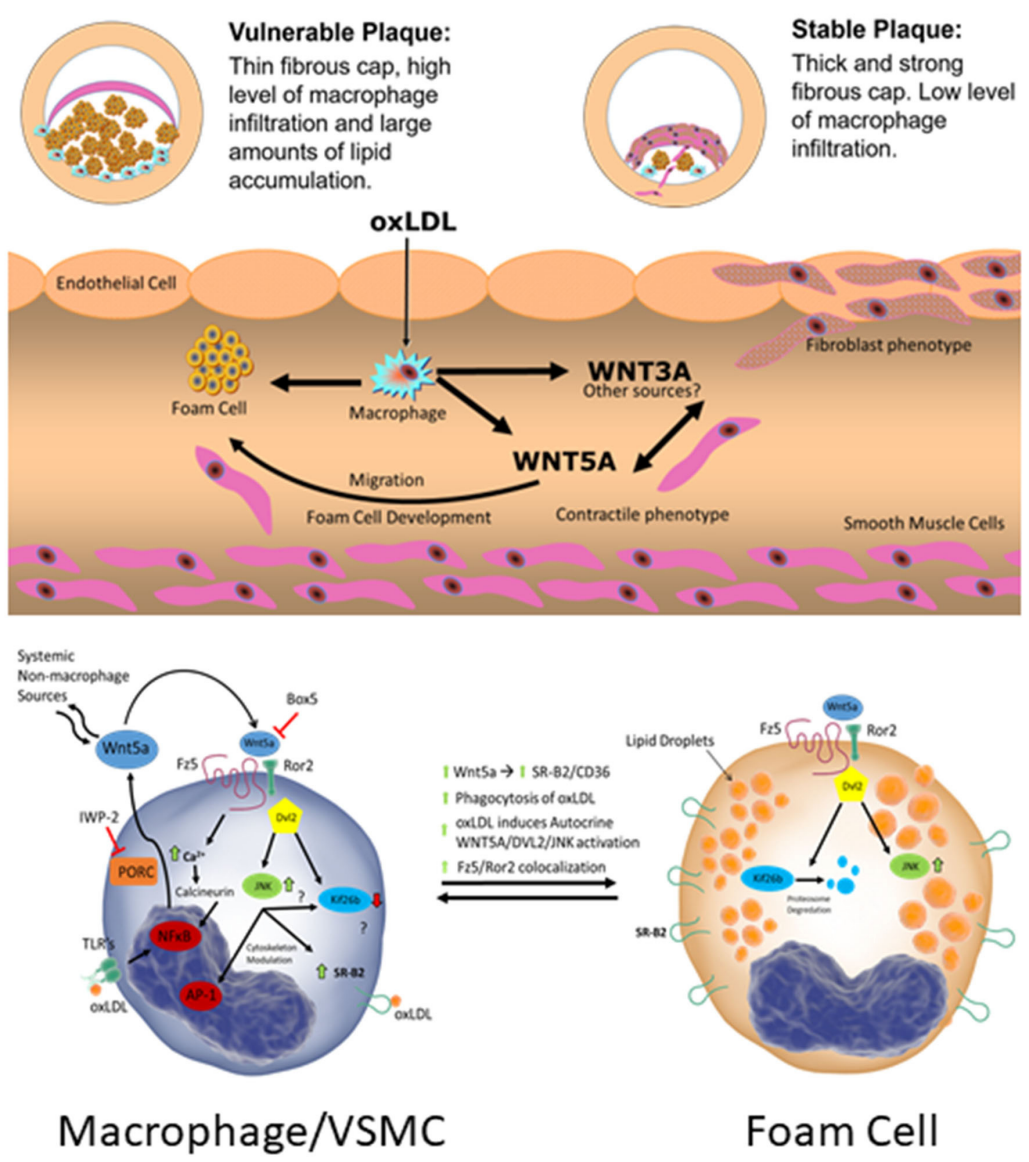


## Introduction

Wnt signaling pathway activation has been reported in many cardiovascular diseases and as a consequence, the modulation of Wnt signaling has aroused scientific interest (1–5). Both non-canonical WNT signaling, as well as canonical signaling have been described to be activated in atherosclerosis (6–8). However, the exact mechanism of non-canonical WNT5A signaling in the atherosclerotic plaque remains elusive. The complex pathogenesis of atherosclerosis involves a cascade of biological events including endothelial dysfunction, monocyte transmigration, vascular smooth muscle cell (VSMC) proliferation and migration, as well as foam cell formation, among others (9). Macrophages and VSMC perform crucial roles at different stages in the pathogenesis of atherosclerosis (9, 10). At the beginning, macrophages and VSMC are associated with the development of foam cells, whereas in advanced stages of the disease, macrophages and VSMC are involved in plaque stability and vulnerability (10, 11).

WNTs primarily act as short-range ligands in autocrine and paracrine signaling, leading to the activation of pathways that regulate diverse cellular biological processes including cell proliferation, cell polarity, cell differentiation, and apoptosis (12). WNT signaling is classified into two general pathways: a canonical pathway involving β-catenin and the non-canonical pathway which is β-catenin independent. The latter consists of two sub-groups: the WNT/Ca^2+^ and the Planar Cell Polarity (PCP) pathways (12, 13). Specific WNT/receptor/co-receptor combinations are particularly important in dictating the resulting downstream signaling effects of a given WNT (12). For example, the co-receptor receptor tyrosine kinase-like orphan receptor 2 (ROR2) is critical for activation of the PCP signaling pathway by WNT5A (14, 15). In this pathway, WNT5A binds to the receptor complex consisting of a Frizzled (FZD) receptor (i.e., FZD5) and co-receptor ROR (i.e., ROR2) (16, 17). Unique combinations of the many receptor/co-receptor/ligand binding often correlate to specific downstream effects by a given WNT ligand (12, 18). Downstream of these receptors, reliable readouts for non-canonical signaling activation have been lacking. Three Disheveled (DVL) proteins are integral to the downstream signaling activation of WNT proteins (14, 15). DVL 1, 2, 3 are not specific to a given pathway; however, DVL2 and c-Jun N-terminal kinase (JNK) are important indicators of non-canonical WNT5A/PCP signaling activation (15, 19). Recently, Susman et al. described that Kinesin Family Member 26B (Kif26b) is rapidly degraded in response to non-canonical WNT signaling activation and is a highly specific marker of non-canonical signaling activation (20, 21).

The interaction between oxidized low-density lipoprotein (oxLDL) and macrophages and VSMC is a critical step in the pathogenesis of atherosclerosis, specifically in the formation of foam cells (22, 23). Foam cells are a critical cell type in atherosclerosis development and VSMC's contribute to a large portion (~50%) of the total foam cell population within the atherosclerotic plaque (23). Activation of macrophages triggers WNT secretion by macrophages and subsequent WNT signaling activation in neighboring cells (24). In this context, our group described increased transcription of WNT5A mRNA in mouse and human macrophages upon exposure to lipopolysaccharide (LPS) and oxLDL (2, 6). Subsequently, we demonstrated that oxLDL increases the expression of CD36 in a FZD5 dependent mechanism (25). CD36 is a key scavenger receptor in macrophages, resulting in lipid uptake and foam cell formation (26). VSMCs also play other important roles in the pathogenesis of atherosclerosis, such as fibrous cap formation. A biological characteristic of VSMCs is their capacity to switch from contractile toward a synthetic phenotype, also referred to as myofibroblast phenotype, involving increased production of extracellular matrix (ECM) and increased proliferation. WNT proteins are implicated in migration and differentiation of VSMCs from the media to the intima which is crucial for fibrous cap formation, an important feature of stable plaques (27–32). These findings demonstrate the importance of macrophages and VSMCs in the atherosclerotic process.

The present study seeks to dissect the effect of oxLDL and WNT5A in THP-1 derived macrophages and a human aortic VSMCs line., Our central hypothesis is that atherogenic stimuli (oxLDL) induce WNT signaling dependent cellular events associated with pathophysiological events in vascular disease.

## Materials and Methods

### *In-vitro* THP-1 and Derived Macrophages Cell Culture

THP-1 cells (Human Monocytic Leukemia cell line) were purchased (American Type Culture Collection, TIB-202™, Manassas, VA), and were cultured in RPMI-1640 supplemented with 10% FBS (complete RPMI) at 37°C, 5% CO_2_ as described previously (6, 33). To induce monocyte-to-macrophage differentiation, THP-1 cells were stimulated with 50 ng/ml Phorbol-12-Myristate-13-Acetate (PMA) for 24 h. After activation and once adherent, the culture medium was removed and replaced with treatment medium (described below). Controls were treated with medium alone plus vehicle where applicable and maintained for the duration of all experiments. At the end of experiments, cell culture medium was collected and centrifuged (900xg) at 4°C for 10 min to remove cell debris. The supernatant was then concentrated 40-fold using Amicon ultra centrifugal filter units (UFC801024, Millipore-Sigma, Burlington, MA). Briefly, supernatant (2 mL) was centrifuged at 7,500xg at 4°C for 10 min (~50 μL final volume). The supernatant was then stored at −80°C before western blotting.

Treatment medium consisted of basal RPMI-1640 (FBS-free) supplemented with rWNT5A (human recombinant WNT5A, 100 ng/mL, R&D Systems, 645-WN, Minneapolis, MN) or low-density lipoproteins (n-LDL and ox-LDL, Alfa Aesar, J65039 & J65591, both 100 μg/mL) for the indicated duration. FZD5 blocking was performed using commercially available Box5 (#681673, Millipore Sigma, Burlington, MA). Cells were pre-treated with Box5 (100 μM in PBS, 1h) before rWNT5A or lipoprotein treatment as indicated. Inhibition of WNT secretion was performed using a commercially available inhibitor of Porcupine (PORCN), Inhibitor of WNT Production-2 (IWP-2, #681671, Millipore Sigma, Burlington, MA). IWP-1 (5 μM in DMSO) was included for 24 h before LDL treatment. DMSO in equal concentration was used as vehicle control in all experiments using IWP-2. Cells were then analyzed using immunofluorescence, confocal microscopy and western blot, described below.

### *In-vitro* Human Aortic Vascular Smooth Muscle Cell Line Culture

Human aortic vascular smooth muscle cells (VSMCs) were purchased from American Type Culture Collection (T/G HA-VSMC, CRL-1999™, Manassas, VA), and were cultured according to the supplier's instructions at 37°C, 5% CO_2_. To make the complete growth medium, the following components (final concentrations) were added to the F-12K base medium (30-2004, ATCC): 0.05 mg/ml ascorbic acid; 0.01 mg/ml insulin (12585014, Gibco); 0.01 mg/ml transferrin (T8158, Sigma, St. Louis, MO); 10 ng/ml sodium selenite (S5261, Sigma) 20% fetal bovine serum; 10 mM HEPES (7365-45-9, American Bioanalytical, Natick, Massachusetts); 10mM TES (T5691, Sigma).

VSMC in passages 2–6 were used for all experiments. Controls were treated with medium alone plus vehicle where applicable and maintained for the duration of all experiments. Treatment medium consisted of basal medium (F-12K Medium, 30-2004, ATCC) supplemented with 100 ng/mL rWNT5A or rWNT3A (human recombinant WNT5A & WNT3A, 645-WN & 5036-WN respectively, R&D Systems, Minneapolis, MN), 10 ng/mL platelet-derived growth factor-BB (PDGF-BB, GF149, Millipore Sigma, Burlington, MA), or 100 μg/mL low-density lipoproteins (n-LDL and ox-LDL, J65039 & J65591, Alfa Aesar) for the indicated duration. Blocking experiments were performed according to the methods described above.

### Migration and Wound Healing Assays

VSMCs were grown in complete growth medium to confluence in 6-well plates and then serum starved (to induce quiescence) for 24 h in serum-free medium (SFM) before treatment. After quiescence/starvation, the cell monolayer was subjected to two parallel scratches on a pre-drawn cross under the plate as a reference point. After the scratch, VSMCs were washed twice with PBS and incubated in 100 ng/mL rWNT5A or rWNT3A supplemented treatment medium. To quantify migration, wells were photographed, including reference points, immediately following wounding and every 6 h for 24 h after wounding to measure the wound area migrated by the VSMCs. Phase-contrast images were acquired on a Nikon Eclipse TS100 microscope. Images were processed using analyzed with ImagePro Plus 7 software (Rockville, MD, USA) as follows: the Lopass large spectral filter (width 5, height 4, passes 3) was applied followed by automatic contrast enhancement (Brightness= 76, Contrast= 67, Gamma= 1.0) and finally threshold into binary output for wound area as a percent of total area. Colored vertical lines (red, blue, black) are estimated wound borders for clarity and do not indicate analyzed border data. Five independent experiments were performed.

### Proliferation Assay

VSMCs were grown in complete growth medium to ~60% confluence and then incubated for 24 h in SFM to induce quiescence. After 24 h, cells were then incubated with treatment medium supplemented with 100 ng/mL rWNT5A, 100 ng/mL rWNT3A or 10 ng/mL PDGF-BB and replenished every 24 h. At the end of the experiments, cell proliferation was assayed using CellTiter 96® AQueous One Solution Cell Proliferation Assay (MTS [(3-(4,5-dimethylthiazol-2-yl)-5-(3-carboxymethoxyphenyl)-2-(4-sulfophenyl)-2H-tetrazolium, salt) assay] (Promega, Madison, WI, USA) according to the manufacturer's instructions. Five independent experiments with each treatment group were performed in triplicate.

### Lipid Accumulation

Neutral lipid droplets were analyzed using Invitrogen's HCS LipidTOX™ Phospholipidosis and Steatosis Detection Kit for image-based high-content screening assays (Invitrogen, H34476, ThermoFisher Scientific, Waltham, Massachusetts). Staining was prepared following the protocol of the manufacturer. Briefly, VSMCs were fixed in formalin and incubated with HCS LipidTOX™ Red Neutral Lipid Stain. Plates were then sealed without washing and imaged. Images were captured on a Nikon Microphot-SA fluorescence microscope or Nikon Eclipse Confocal Microscope A1 Ti-E (images were recorded at 512 × 512 or 2,048 × 2,048 pixels with a pixel dwell of 3.1) using Nikon NIS-Elements software (Nikon Melville, NY). At least ten high-power fields per biological replicate were collected. Three independent experiments were performed.

### Immunofluorescence and Confocal Microscopy

THP-1 cells were cultured on glass coverslips in 12-well plates. At the endpoint of all experiments, cells were washed twice with PBS, then fixed in ice-cold methanol for 10 min and subsequently washed three times with ice-cold PBS. The cells were then blocked with 1% BSA in PBST (PBS + 0.1% Tween 20) for 30 min. Cells were then incubated overnight at 4°C in antibodies against FZD5 and ROR2 (described in the Immunochemistry section) diluted in 1% BSA-PBST. On separate coverslips, species-matched IgG at the same concentration was used as an isotype control for the primary antibodies. After overnight incubation, the primary antibodies were carefully aspirated, and the cells were washed three times with PBS for 5 min each wash. The cells were then incubated with species-matched secondary antibody (1/1,500, A11008, A11005, Invitrogen) diluted in 1% BSA-PBS at room temperature for 1.5 h. The secondary antibody solution was then decanted and cells washed three times with PBS, 5 min each, in the dark. Coverslips were counterstained with DAPI and mounted on glass slides. Images were captured on a Nikon Eclipse Confocal Microscope A1 Ti-E (images were recorded at 512 × 512 and 2,048 × 2,048 pixels with a pixel dwell of 3.1) using Nikon NIS-Elements software. Experiments were performed in triplicate with at least 10 images per biological replicated captured at 20x, 60x, and 100x. For each experiment and biological replicate, over 100 cells were evaluated.

### Western Blot

Experiments were performed on THP-1 derived macrophages and VSMCs as described above (25). Total proteins were extracted using Cell Lysis Buffer (Cell Signaling Technology, Danvers, MA) and Complete EDTA-Free Protease and Phosphatase Inhibitors (Roche, Basel, Switzerland). Protein concentrations from the cell lysates were determined using the bicinchoninic acid assay (BCA) kit (Pierce, Rockford, IL). Equal protein (30 μg) was loaded on 4–12% Bis-Tris gels (Invitrogen, #NP0322BOX) and transferred to polyvinylidene fluoride (PVDF) membranes (ThermoFisher Scientific, 88518), blocked in 5% milk, and probed overnight with specific primary antibodies diluted in 5% BSA in 0.1% TBS-Tween. Primary antibodies included rabbit anti-human PDGF-receptor beta (1/40,000, ab32570, Abcam, Cambridge, UK), rabbit anti-human β-catenin (1/40,000, ab32572, Abcam), rabbit anti-human non-phospho (Active) β-catenin (Ser33/37/Thr41) (1/40,000, #8814 Cell Signaling Technology, Danvers, MA), rabbit anti-human Collagen I (1/40,000, ab138492, Abcam), mouse anti-human Smoothelin (1/40,000, ab8969, Abcam), rabbit anti-human Calponin (1/40,000, ab46794, Abcam), rabbit anti-human CD36 (1/40,000, ab133625, Abcam), rabbit anti-human alpha smooth muscle actin (1/400,000, ab124964, Abcam), rabbit anti-human Disheveled 2 (DVL2) (1/20,000, 3216s, Cell Signaling Technology), Rabbit anti-human CD36 (1/40,000, ab133625 Abcam), Rabbit anti-human Phospho-SAPK/JNK (Thr183/Tyr185) (1:10,000, 9251, Cell Signaling Technology), Rabbit anti-human JNK (1:40,000, 9258, Cell Signaling Technology), mouse anti-human WNT5A (1:180,000, MAB15432, Abnova, Taipei City, Taiwan), rabbit anti-human Kif26b (1:20,000, generously provided by Dr. Henry Ho) (20), mouse anti-human WNT3A (1:40,000, ab81614 Abcam) with mouse anti-human alpha-tubulin (1/100,000, #3873, Cell Signaling Technology) or rabbit anti-human GAPDH (1/80,000, #3683, Cell Signaling Technology) as loading control. JNK proteins (phosphorylated and total) were evaluated on separate membranes and probed on the same day. After overnight incubation at 4°C, membranes were washed and exposed to appropriate HRP conjugated secondary for 1 h (7074s or 7076s, Cell Signaling Technology). Enhanced chemiluminescence reagent (GE Healthcare, Chicago, IL) was used to detect antibody binding and visualized upon exposure to autoradiographic film. All experiments were performed in at least triplicate unless otherwise indicated.

### Statistical Analysis

GraphPad Prism5 software was used to generate graphs and for statistical analysis. For MTS proliferation data, one-way analysis of variance (ANOVA) was used to determine differences between groups followed by Tukey *post-hoc* analyses. Lipid accumulation analysis is described above. Migration analysis was performed using a two-way analysis of variance (ANOVA) to determine differences between groups at each time-point followed by Tukey's post-test to compare means. For densitometric analysis of western blots, one-way ANOVA was used to compare means of groups normalized by loading controls. For all analyses, *p* < 0.05 was considered statistically significant. All experiments were repeated in triplicate unless otherwise indicated.

## Results

### oxLDL Regulates WNT5A Expression and Signaling Activation in THP-1 Derived Macrophages and Human Aortic Vascular Smooth Muscle Cells

**Figure 1:**
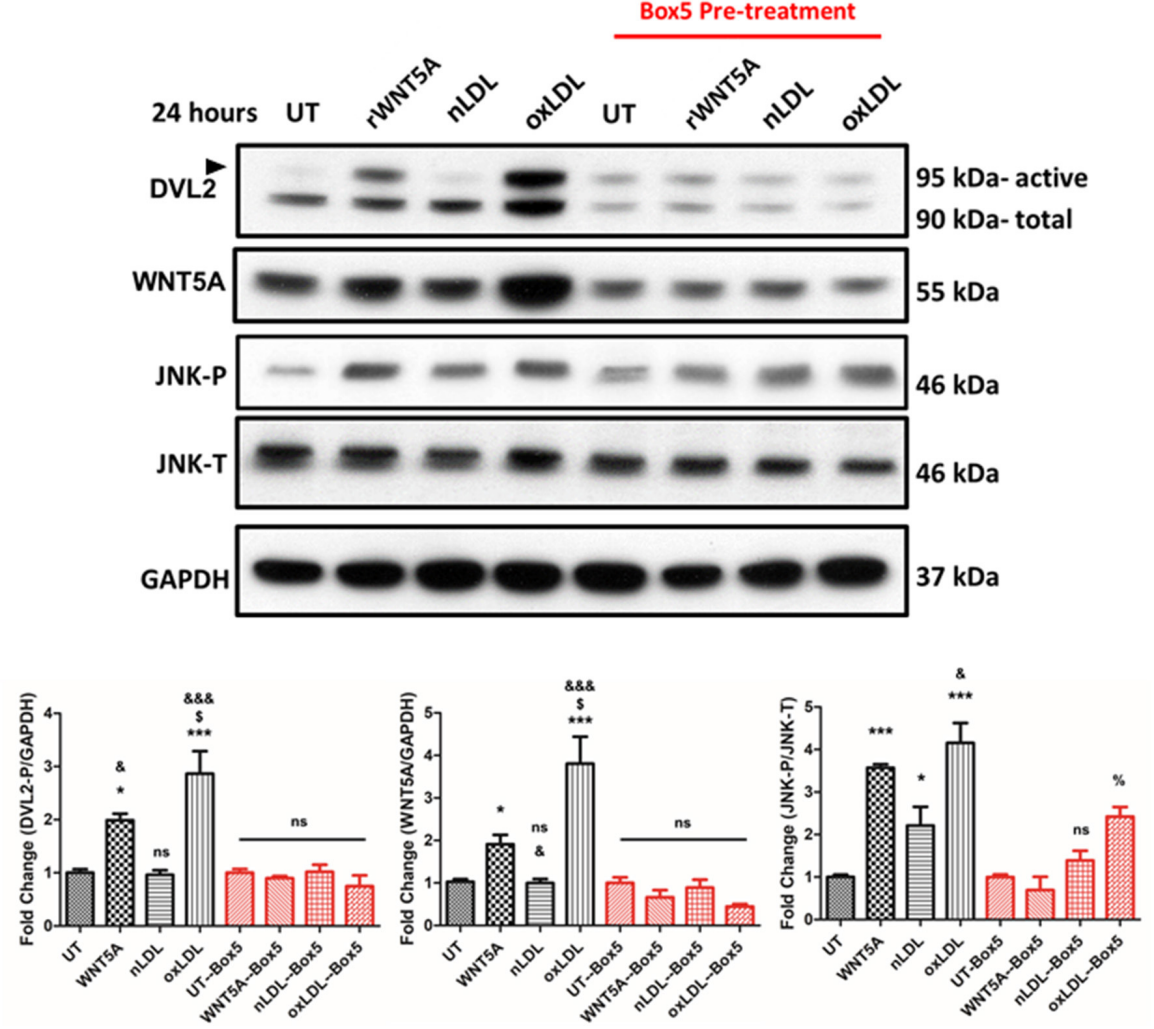
Box5 Blocks rWNT5A and oxLDL-Mediated Non-canonical Wnt Signaling in THP-1-Derived Macrophages. Representative western blots of THP-1-derived macrophages. THP-1-derived macrophages were pre-treated, or not, with Box5 (100 mM) for 1 h or not followed by treatment with rWNT5A (100 ng/mL), nLDL, or oxLDL (both 100 μg/mL) for an additional 24 h. Box5 significantly inhibited rWNT5A-induced and oxLDL-induced DVL2 activation (black arrow). Box5 also inhibited rWNT5A-induced JNK activation but not oxLDL-induced JNK activation, although overall activation was reduced. Experiments were performed in triplicate and western blot band intensities were quantified and are presented as bar graphs (mean ± SEM) of the ratios of DVL2-p/GAPDH, WNT5A/GAPDH, JNK-P/JNK-T expression relative to respective controls (UT or UT-Box5). ns = not significant compared to untreated control (UT or UT-Box5) (*p* > 0.05);^*^ = compared to UT control (*p* < 0.05);^***^ = compared to UT control (*p* < 0.001); % = compared to Box5 UT control (*p* < 0.05); & = compared to nLDL control (*p* < 0.05); &&& = compared to nLDL control (*p* < 0.001). $ = compared to rWNT5A (*p* < 0.05).

As an approach to investigate the role of oxLDL on WNT signaling in macrophages, THP-1 derived macrophages were used. Results suggest that oxLDL induces WNT5A expression and DVL2 activation in THP-1 derived macrophages (Figure 1). To investigate the mechanism, we inhibited at the receptor level (FZD5) and inhibited endogenous WNT secretion.

**Figure 2:**
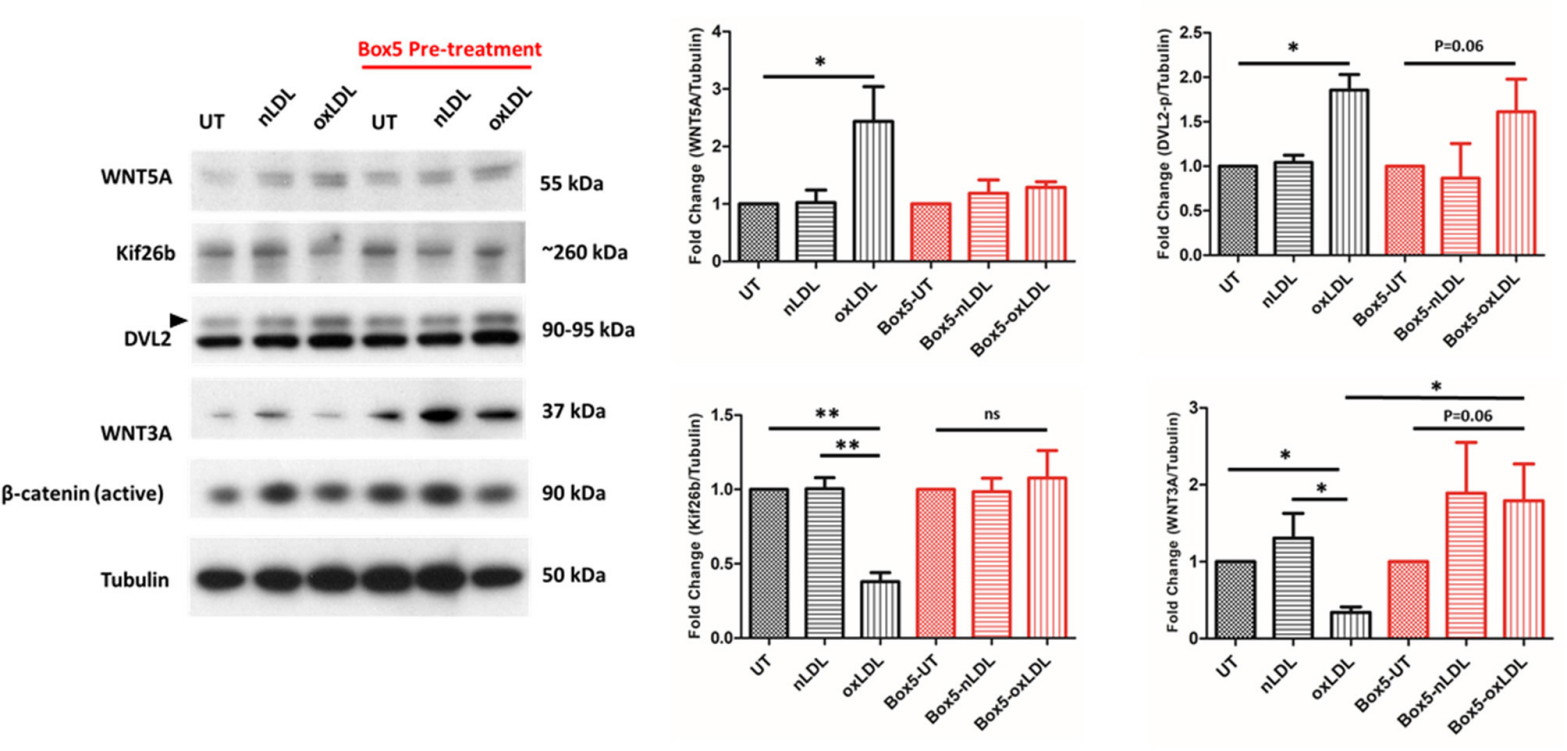
Box5 Blocks rWNT5A and oxLDL Mediated Non-canonical Wnt Signaling in Human Vascular Smooth Muscle Cell Line. Representative western blots evaluating the expression of WNT signaling components in response to oxLDL. In response to oxLDL, there was a significant increase in WNT5A and DVL2 (black arrow) expression (*p* < 0.05, *p* < 0.01; respectively). There was also a significant decrease in Kif26b expression (*p* < 0.01), indicative of activation of non-canonical WNT signaling in oxLDL-treated VSMCs. There was a significant decrease in WNT3A expression (*p* < 0.01), in the oxLDL treated group. An overall increasing trend in WNT3A and active β-catenin expression in the Box5 pre-treated groups was found. All protein experiments were performed in triplicate and western blot band intensities were quantified and are presented as bar graphs (mean ± SEM) of the ratio of control normalized (protein of interest)/tubulin protein. ns = not significant to untreated control (UT or UT-Box5); * = compared between groups (*p* < 0.05); **= compared between groups (*p* < 0.01).

First, FZD5 signaling was inhibited using the FZD5specific inhibitor Box5. Box5 has been described to be a competitive inhibitor of WNT5A through binding FZD5 receptor (34, 35). The results in Figure 1 show that Box5 pre-treatment inhibited rWNT5A induced DVL2 activation andoxLDL-induced DVL2 activation in THP-1 derived macrophages (Figure 1). Further downstream in the WNT signaling pathway, rWNT5A and oxLDL increased activation of JNK, suggesting non-canonical WNT/PCP pathway activation. In the VSMCs line studied, oxLDL also significantly increased WNT5A expression and DVL2 activation (Figure 2, both *p* < 0.05), bul also Box5 pre-treatment inhibited WNT5A signaling activation. Interestingly, oxLDL significantly decreased WNT3A expression in VSMCs and a reduction in active β-catenin relative to UT control (Figure 2, *p* < 0.01). Kif26b was significantly decreased in response to oxLDL compared to UT and nLDL (both, *p* < 0.01), confirming an upregulation of non-canonical WNT signaling.

**Figure 3:**
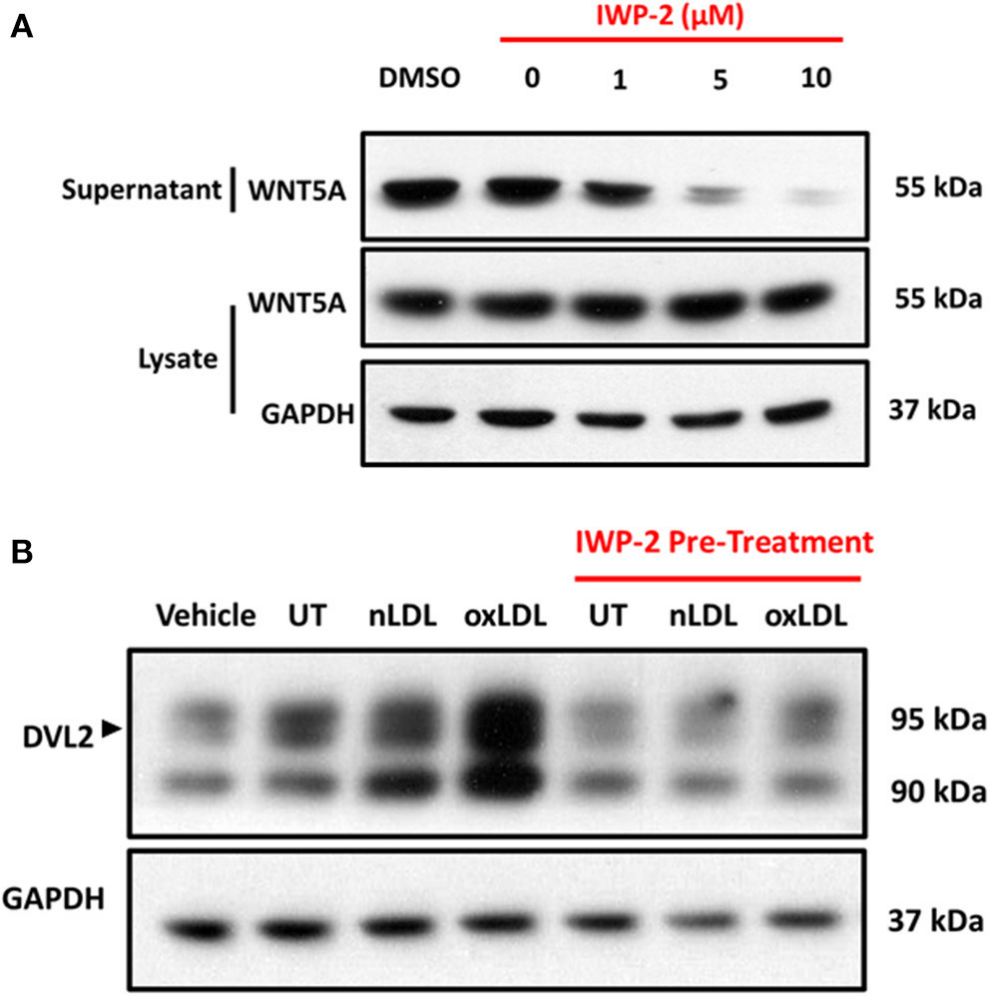
oxLDL-Induced DVL2 Activation is Dependent on WNT Secretion. **(A)** Representative western blots of THP-1-derived macrophages pre-treated with IWP-2 for 24 h at indicated concentrations. Preparation of THP-1 culture medium for western blot was described in the material and methods. In addition to protein estimation by bicinchoninic acid assay (BCA assay), Coomassie staining was used to confirm equal gel loading. IWP-2 demonstrated a concentration-dependent inhibition of WNT5A secretion into the cell culture supernatant, with no appreciable change in the intracellular content detected in whole-cell lysates. **(B)** Representative western blots of THP-1-derived macrophages pre-treated with vehicle (DMSO) or IWP-2 (5 μM) for 24 h and then treated with nLDL, or oxLDL (both 100 μg/mL) for an additional 24 h. IWP-2 inhibited oxLDL-induced DVL2 activation (black arrow), as evidenced by the reduction in intensity and electrophoretic mobility shift from ~90 to ~95 kDa. All experiments were performed in triplicate.

Second, endogenous WNT secretion was inhibited to investigate if oxLDL-induced DVL2 activation was specific to the WNT signaling pathway, or an independent effect of oxLDL. To accomplish this, we used the inhibitor of Wnt Production 2 (IWP-2). In THP-1 derived macrophages there was reduced WNT5A detection in the cell culture supernatant after treatment with increasing concentration of IWP-2, with no changes in WNT5A expression in the whole-cell lysate (Figure 3A). We then evaluated the intracellular activation of DVL2 in cells pre-treated for 24 h with IWP-2 (5 μM DMSO) and then treated with nLDL or oxLDL. Compared to control cells, there was a clear decrease in DVL2 activation in response to oxLDL in the IWP-2 group (Figure 3B). Supplementary Figure 1 shows that WNT5A activated DVL2 over time, as evidenced by the electrophoretic mobility shift from ~90 to 95 kDa. Together, these results demonstrate that oxLDL-induced DVL2 activation requires intact WNT secretion.

### WNT5A Regulates THP-1 Derived Macrophages and VSMC-Derived Foam Cell Formation

**Figure 4:**
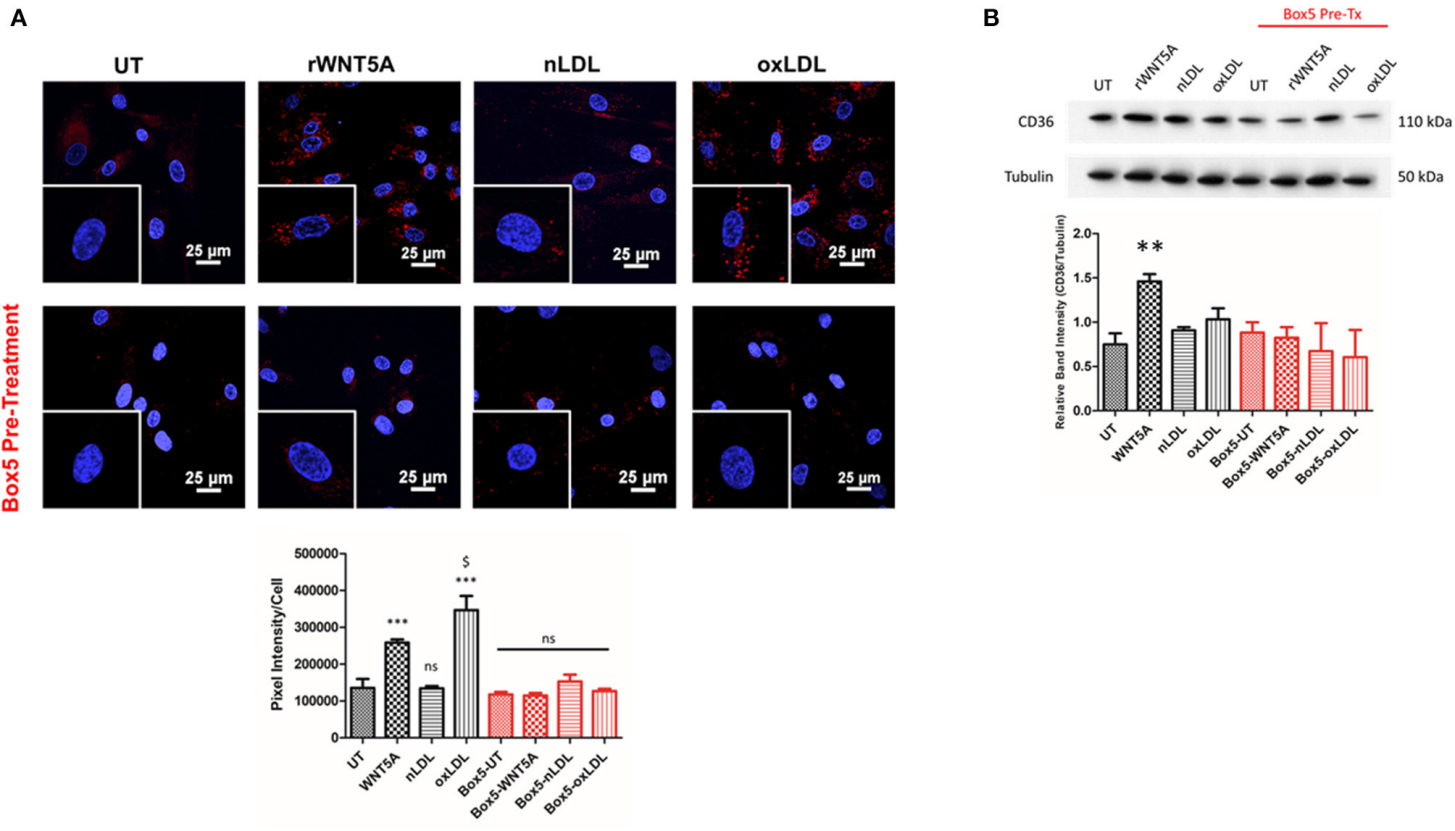
WNT Proteins Regulate Lipid Accumulation in THP-1 Macrophages and VSMCs. **(A)** LipidTOX™ staining was used to evaluate the accumulation of neutral lipids within VSMCs. Representative 60x confocal images are shown. Equally zoomed insets are shown in the bottom left-hand corner of each image. The untreated (UT) controls were held for the duration of all *in vitro* experiments. Compared to UT and nLDL groups, cells treated with rWNT5A (100 ng/ml) and oxLDL (100 μg/mL) have increased red staining in the cytoplasm, indicating an increase in lipid within the cell. Box5 pre-treatment completely blocked lipid accumulation in response to rWNT5A and oxLDL. Quantification of lipid accumulation in each treatment group is representative of two independent experiments. **(B)** Western blots evaluating the expression of CD36 in VSMCs are shown. In response to rWNT5A (100 ng/mL), expression of CD36 in VSMCs was significantly increased (**, *p* < 0.01), which was blocked by pre-treatment with Box5. *** = compared to UT control (*p* < 0.001); $ = compared to rWNT5A (*p* < 0.05).

To confirm that oxLDL results in lipid accumulation and foam cell development, THP-1 derived macrophages were treated with 100 μg/mL nLDL or oxLDL and stained with LipidTox Red and Oil Red O. Results demonstrated significant lipid accumulation in macrophages treated with oxLDL (Supplementary Figure 1). LipidTox staining of VSMCs treated with rWNT5A or oxLDL also resulted in a strong increase in lipid droplet accumulation within the cytoplasm (Figure 4A). When VSMCs were pre-treated with Box5, the lipid accumulation in response to WNT5A and oxLDL was significantly blocked (Figure 4A, *p* < 0.001). Further, VSMCs increase the expression of CD36 increased in VSMCs in response to rWNT5A (Figure 4B, *p* < 0.01). Importantly, this increase was also blocked by Box5, suggesting a FZD5-specific mechanism for lipid accumulation.

The role of canonical signaling in foam cell formation is unknown. Our results demonstrated that WNT3A decreased lipid droplets in macrophages (Supplementary Figure 3). There was an increase in lipid accumulation in the WNT5A-treated group, confirming our previous report (Supplementary Figure 3). In the same experiment, macrophages were treated with WNT5A/WNT3A concurrently (both 100 ng/mL) to evaluate if there would be any opposing effects. WNT5A/WNT3A co-treatment demonstrated a similar level of lipid accumulation as untreated (UT) cells, which suggested opposing effects of WNT5A/WNT3A in regulating lipid droplet accumulation.

### rWNT5A and oxLDL Promote FZD5 and ROR2 Co-localization *in-vitro* in THP-1 Derived Macrophages

**Figure 5:**
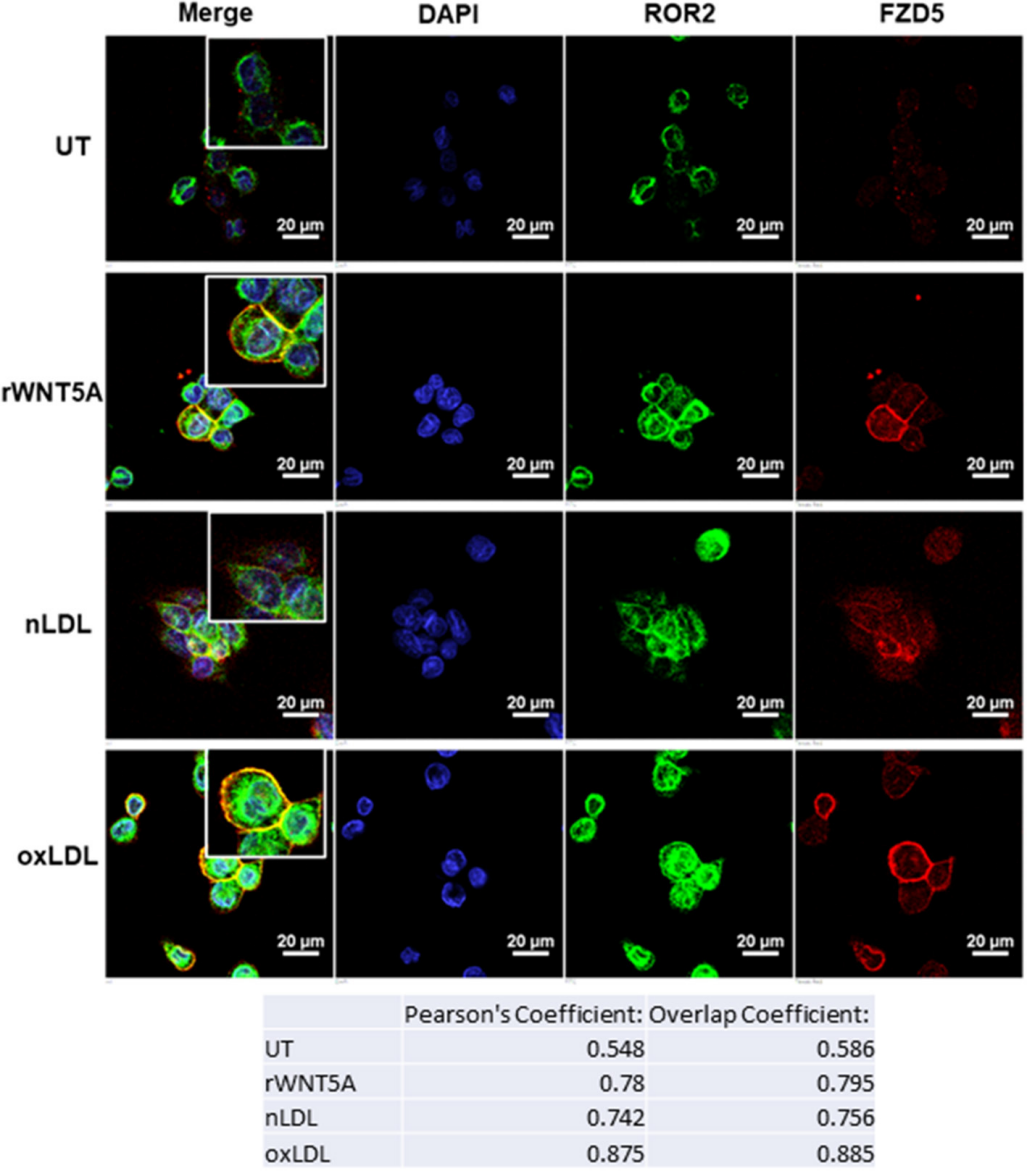
WNT5A Receptors, FZD5 and ROR2, are Co-localized *in vitro* in Response to oxLDL and WNT5A. Representative confocal immunofluorescence (IF) images of THP-1-derived macrophages. Cells were differentiated with PMA for 24 h and then treated with rWNT5A (100 ng/mL), (nLDL) or oxLDL (both 100 μg/mL) for an additional 24 h. Cells were then fixed, dual-stained with FZD5 (Red) and ROR2 (Green), and counterstained with 4′,6-diamidino-2-phenylindole (DAPI). Compared to untreated cells (UT), rWNT5A increased the co-localization of the FZD5 and ROR2 receptors at the plasma membrane (Pearson's Correlation: UT = 0.548, rWNT5A = 0.78). Further, oxLDL-treated cells showed an even greater increase in co-localization of the FZD5 and ROR2 receptors compared to all groups (Pearson's Correlation: oxLDL = 0.875). Immunofluorescent images are at 60x with equally zoomed insets for clarity (scale bar= 20 μm). Experiments were performed in duplicate and repeated three independent times. Representative negative control and isotype control images are included in the data supplement (Supplementary Figure 2).

Receptor availability and localization are important for ligand-mediated signaling cascades. We next investigated the mechanism of WNT5A and oxLDL in the co-localization of FZD5 and ROR2 receptors *in-vitro*. Figure 5 demonstrates the expression of these receptors in THP-1derived macrophages in response to rWNT5A (100 ng/mL), nLDL (100 μg/mL), and oxLDL (100 μg/mL) for 24 h. Results in Figure 5 show a strong increase in expression of FZD5 and ROR2 in response to rWNT5A, nLDL, and oxLDL. Importantly, there was strong co-localization of these receptors at the cellular membrane in response to WNT5A and oxLDL (Figure 5, Pearson's Coefficients of *r* = 0.78 and *r* = 0.875, respectively). Isotype and negative controls are available in the data supplement (Supplementary Figure 2). These results provide evidence to suggest that in response to oxLDL non-canonical WNT5A signaling was activated.

### WNT5A and WNT3A Differentially Regulate VSMC Migration and Proliferation

**Figure 6:**
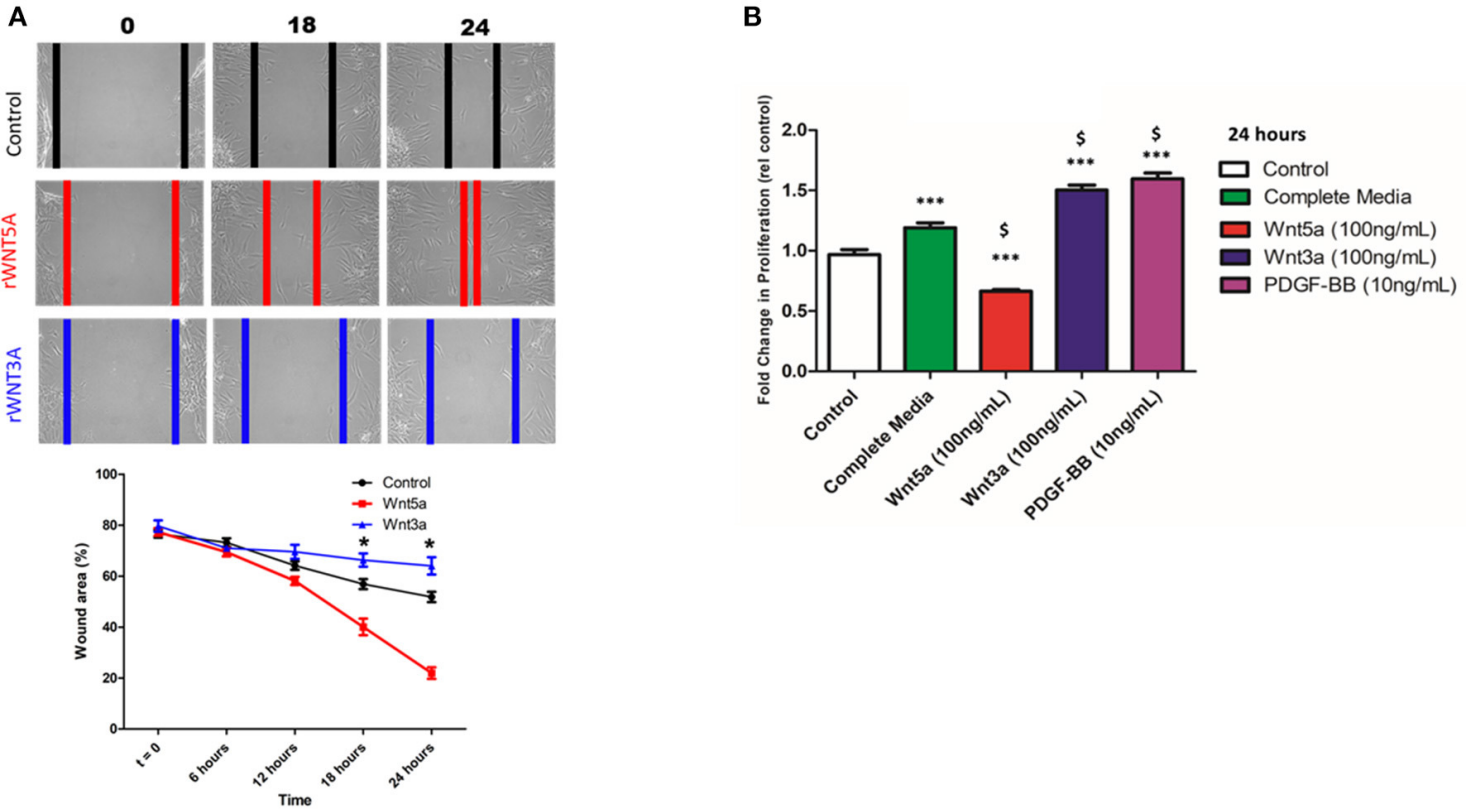
WNT Proteins Differentially Regulate VSMC Migration and Proliferation. **(A)** Representative phase-contrast microscopy images of migrating VSMCs in response to rWNT5A or rWNT3A. Cells were grown to confluence and subjected to wound-healing scratch assay. After the scratch, VSMC were washed 2x with PBS and incubated in rWNT5A or rWNT3A supplemented medium (both 100 ng/mL). VSMCs were then imaged every 6 h. Colored vertical lines are the estimated wound borders for clarity. Quantification was performed on phase-contrast images. rWNT5A promoted migration in comparison to untreated control (UT), as evidenced by a significant decrease in % wound area (*p < 0.01, *n* = 5 experiments). Additionally, rWNT3A inhibited migration compared to UT (*p < 0.01, *n* = 5 experiments). **(B)** Results of MTS proliferation assays in VSMCs. Cells were seeded at ~60% confluence then incubated for 24 h in basal medium to induce quiescence. After 24 h, cells were then incubated with the indicated treatment medium. Date were normalized to a standard curve to determine cell number and represented as fold change. rWNT5A inhibited cell proliferation relative to control and complete medium, whereas rWNT3A induced VSMC proliferation almost to the same degree as the positive control (PDGF-BB). Data are representative of five independent experiments with each group performed in triplicate, $ *p* < 0.05 relative to complete medium; ***p < 0.001; ns = not significant relative to control for each time point.

A wound healing/scratch assay was used to evaluate the migration *in vitro* of human VSMC cell line in response to rWNT5A or rWNT3A. Figure 6A showed representative phase-contrast microscopy images of migrating VSMCs in response to rWNT5A or rWNT3A with approximated migrating border (red, blue, black) for clarity. Quantification of migration demonstrated that, in response to rWNT5A (100 ng/mL), VSMCs migrated significantly more compared to control cells (18 and 24 h, *p* < 0.01) (Figure 6A). Further, in response to rWNT3A (100 ng/mL), VSMC migration was reduced relative to control (18 and 24 h, *p* < 0.01) (Figure 6A). These results suggested contrasting roles for WNT5A and WNT3A responses in VSMC migratory capacity.

Proliferation of VSMCs in response to rWNT5A and rWNT3A was evaluated using MTS [(3-(4,5-dimethylthiazol-2-yl)-5-(3-carboxymethoxyphenyl)-2-(4-sulfophenyl)-2H-tetrazolium, salt] assay. rWNT5A inhibited cell proliferation relative to control and complete medium, whereas rWNT3A induced VSMC proliferation to a similar magnitude as a positive control (PDGF-BB) (Figure 6B). These results suggested distinct functions of WNT5A and WNT3A in VSMC proliferation.

### WNT5A and WNT3A Perform Contrasting Functions in Determining Phenotypic Markers in the Human VSMC Line Studied

**Figure 7:**
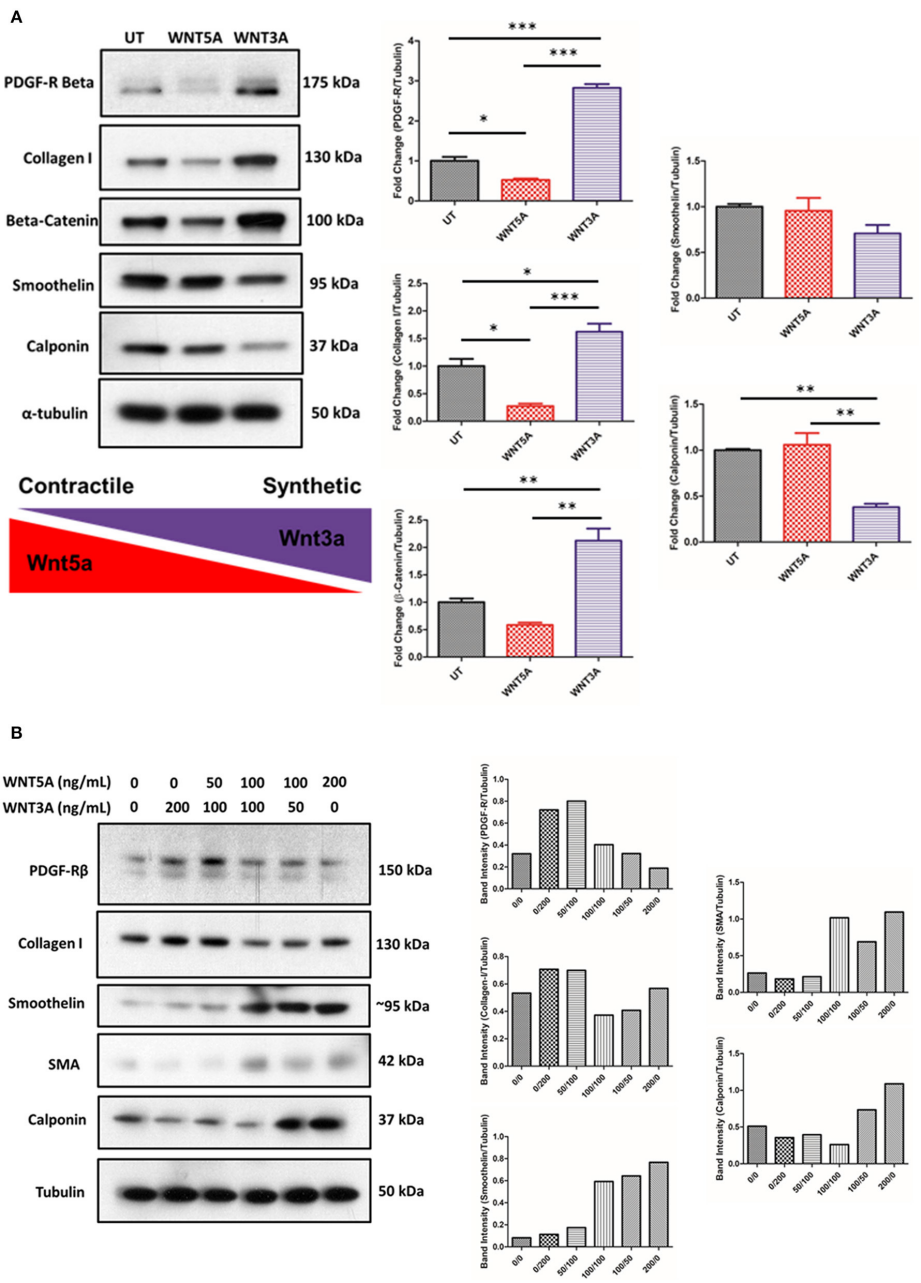
rWNT5A and rWNT3A Regulate VSMC Phenotypic Switching. Representative western blots evaluating the expression of markers of synthetic or contractile VSMCs. VSMCs were incubated in rWNT5A or rWNT3A supplemented medium (both 100 ng/mL) for 24 h. **(A)** rWNT3A increased expression of PDGF-receptor-beta, collagen I, and β-catenin. rWNT3A decreased markers of contractile phenotypic differentiation (calponin, smoothelin). rWNT5A did not significantly reduce contractile markers. rWNT5A decreased PDGF-receptor-beta and markers of synthetic phenotypic differentiation (collagen I). rWNT5A also decreased β-catenin expression. Experiments were performed in triplicate. **(B)** Representative western blots evaluating expression markers of synthetic or contractile VSMCs. VSMCs were incubated in rWNT5A or rWNT3A in combination at the indicated concentrations (0, 50, 100, 200 ng/mL for each WNT) for 24 h. Taken together, these results suggest that under different concentrations of rWNT5A and rWNT3A, VSMCs up-regulate or down-regulate expression of the assayed phenotypic markers. Data are representative of one experiment performed in duplicate. Western blot band intensities were quantified and are presented as bar graphs (mean ± SEM) as band intensity or ratio of control normalized (protein of interest)/Tubulin. * = *p* < 0.05; ** = *p* < 0.01; *** = *p* < 0.001 significant difference between groups.

VSMCs were incubated in medium supplemented with 100 ng/mL rWNT5A or rWNT3A for 24 h. Protein lysates were then isolated and assayed by western blot. Results demonstrated that rWNT3A significantly increased the expression of platelet-derived growth factor receptor beta (PDGF-R-β) and β-catenin (Figure 7A, *p* < 0.001 and *p* < 0.01, respectively). Figure 7A also demonstrated that rWNT3A decreased markers of contractile phenotypic differentiation (calponin, *p* = 0.01 and smoothelin, *p* = ns). Concomitantly, rWNT3A increased expression of the synthetic marker, collagen I (*p* < 0.05). Conversely, rWNT5A decreased (*p* < 0.05) collagen I expression (*p* < 0.05). Furthermore, rWNT5A decreased detection of β-catenin, which was significantly different from WNT3A (*p* < 0.01), and rWNT3A significantly increased β-catenin expression relative to control (*p* < 0.01), confirming significant signaling crosstalk of these two WNT proteins (Figure 7A). We next wanted to evaluate if these WNT proteins, given their apparent opposing effects, directly competed to regulate VSMC phenotypic markers.

To determine if WNT signaling pathways interacted, we first chose to test if archetypal WNTs, WNT5A, and WNT3A, interact to determine VSMC phenotypic markers. VSMCs were incubated for 24 h with varying concentrations of recombinant WNT5A/3A proteins. Intriguingly, we found a clear WNT concentration-dependent gradient of the VSMC phenotypic markers investigated (Figure 7B). In summary, under higher relative concentrations of WNT5A, VSMC's may be directed toward a more contractile/quiescent phenotype, whereas with higher relative concentrations of WNT3A, VSMCs may be induced toward a synthetic, proliferative phenotype.

## Discussion

The interaction between oxLDL, macrophages, and VSMC are critical in the pathogenesis of atherosclerosis, specifically during atheroma formation. In a previous study, our group demonstrated that human peripheral blood macrophages increase transcription of WNT5A mRNA upon exposure to oxLDL (6). More recently, we have reported that WNT5A increases expression of CD36, resulting in oxLDL/lipid uptake within macrophages (25). The results presented in this study expand on this previous work. Our data suggest that WNT5A signaling activation through the FZD5 receptor is a potencial mechanism involved in foam cell formation in THP-1 derived macrophages and human aortic VSMC cell line used in this study. Our results cannot rule out the effects of blocking alternative FZD5 ligands; however, WNT5A, via FZD5 binding, remains an attractive target for therapeutic intervention.

WNT signaling is a complex system of multiple pathways, and the understanding of non-canonical WNT signaling is less well-described. In this study, we investigated the involvement of non-canonical WNT signaling pathways using Kif26b as a specific marker of non-canonical WNT signaling activation (20). Kif26b is specifically targeted by ubiquitination and degradation in a non-canonical WNT signaling-specific manner. Using this approach, we confirmed activation of non-canonical WNT5A signaling activation in VSMCs upon treatment with oxLDL. oxLDL also promotes WNT5A protein expression and secretion in THP-1 macrophages. Thus, non-canonical WNT signaling likely plays a role in arterial wall vascular biology.

WNT signaling activation is dependent on the receptor and cell context; here, we studied two receptors involved in non-canonical WNT signaling, FZD5 and ROR2 (36). Our results showed a high degree of FZD5 and ROR2 co-localization in foam cells *in vitro*. FZD5/ROR2 co-localization was inducible in response to oxLDL and WNT5A; interestingly, this effect was more pronounced with oxLDL treatment. Additionally, our results demonstrated for the first time that oxLDL-induced activation of WNT5A/DVL2 signaling was dependent on WNT5A secretion, suggesting an autocrine, positive-feedback loop (oxLDL/WNT5A/DVL2) where WNT5A signaling was activated in response to oxLDL. Dissecting further down in the WNT signaling pathway, our results showed that oxLDL-induced JNK-p46 activation was only partially blocked by Box5, indicating that oxLDL was likely inducing JNK activation via a secondary pathway independent of FZD5. This result is in line with results reported by Kim et al. where they demonstrated WNT5A induces JNK and NF-κB activation in THP-1 cells (37).

Although evidence has implicated non-canonical WNT signaling as pro-atherogenic (4, 6, 25), numerous reports demonstrated a critical role of the canonical WNT signaling pathway in cardiovascular disease (38–40). Other studies support the concept of an intricate balance of canonical vs. non-canonical WNT signaling pathways and their importance in vascular biology (41, 42). VSMC phenotypic switching was described as an important initiating factor and key pathophysiological change in proliferative vascular diseases (43, 44). VSMCs have a high capacity to differentiate/de-differentiate from a contractile to a synthetic phenotype, as evidenced by changes in protein markers in response to physical or molecular stimuli from the microenvironment. VSMC phenotypic differentiation involves proliferation, migration, and extracellular matrix (ECM) deposition. The current study showed the effect of WNT signaling on VSMC phenotypic markers. Interestingly, our results showan increase in collagen I protein in response to rWNT3A; this result is in line with previous reports that demonstrated increases collagen I RNA in mouse VSMCs line CRL-2797 in response to WNT3A (5). Further, our data demonstrated that archetypal canonical and non-canonical WNT proteins (WNT3A and WNT5A, respectively) induced proliferation (WNT3A) or migration (WNT5A). Co-treatment experiments demonstrated that the effects of WNT5A and WNT3A can be reversed in a concentration-dependent manner. The phenotypic switching of VSMCs has been associated to fibrous cap thickness, which dictated plaque vulnerability (8, 31, 32). Previous reports in the literature also regard VSMCs as important contributors to the total foam cell population (23). In line with this, our results also show that WNT5A may promote differentiation of thehuman VSMCs line used in the study into foam cells. All of these results suggest that WNT signaling plays a role in vascular smooth muscle cell biology. A limitation of our study is that our *in vitro* models used immortalized cell lines, these studies need to be extended using different models to comprehensively evaluate these novel mechanisms in humans.

In summary, this study implicated WNT5A signaling in THP-1 derived macrophages, human aortic VSMCs, and foam cells with the following lines of evidence. First, oxLDL promoted FZD5 and ROR2 co-localization in THP-1 derived macrophages, providing crucial novel information regarding WNT/FZD5 receptor/co-receptor combinations in foam cells. Further, oxLDL-induced DVL2 activation was dependent on WNT secretion, demonstrating for the first time a mechanism whereby WNT5A signaling was activated in response to oxLDL. Results also suggested that a balance of canonical/non-canonical WNT signaling activation may regulate switching the VSMCs phenotype. Taken together, these results suggest that WNT signaling overall may be an important player in thepathophysiology of vascular disease, making modulation of WNT signaling a potential therapeutic tool to improve cardiovascular disease outcomes.

## Data Availability Statement

The raw data supporting the conclusions of this article will be made available by the authors, without undue reservation.

## Author Contributions

IA, CS, and RM designed the study and prepared the manuscript, with input from all authors. IA and CS performed experiments and data analyses. MS and RM guided the interpretation of the results. All authors contributed to the article and approved the submitted version.

## Conflict of Interest

The authors declare that the research was conducted in the absence of any commercial or financial relationships that could be construed as a potential conflict of interest.

## Supplementary Material

Supplementary Figure 1 Foam Cell Development and WNT5A Signaling. Representative Oil Red O-stained images of THP-1-derived macrophages. Cells were differentiated with PMA for 24 h and then treated with control cholesterol (nLDL) or oxidized cholesterol (oxLDL) (both 100 μg/mL) for an additional 24 h. Cells were then imaged by light microscopy. The same treatment experiment was performed using a different detection system. Invitrogen's HCS LipidTOX Phospholipidosis and Steatosis Detection Kit for image-based high content screening assays [ThermoFisher Scientific (Invitrogen, H34476), Waltham, MA, USA] were used previously in our lab. Staining was prepared following the manufacturer's protocol. Western blotting was used to evaluate the activation of WNT5A signaling pathway activation in response to rWNT5A. Macrophages were differentiated as described in the Materials and Methods section. Compared to untreated (UT), macrophages treated with rWNT5A (100 ng/ml) had significant DVL2 activation (black arrow), evidenced by the electrophoretic mobility shift of the band from ~90 to ~95 kDa which has been previously described (15). DVL2 activation can be appreciated in 30 min of rWNT5norA treatment and sustained to 6 h. JNK was also activated over the same time frame. Experiments were performed in triplicate and western blot band intensities were quantified and are presented as bar graphs (mean ± SEM) of the ratio of JNK-P/JNK-T and DVL2/GAPDH expression relative to control. Quantification of band pixel intensity was determined by ImageJ software from three independent experiments. ns = *p* > 0.05, ^*^*p* < 0.05, ^**^*p* < 0.01, ^***^*p* < 0.001.

Supplementary Figure 2 Co-localization in Macrophage Culture. Representative confocal fluorescent images of THP-1-derived macrophages. Cells were treated as described in the Materials and Methods section. On separate coverslips, species-matched IgG at the same concentration was used as isotype control and no primary antibody was used as negative control; cells were counterstained with 4′,6-diamidino-2-phenylindole (DAPI) and images are at 600x (scale bar = 50 μm).

Supplementary Figure 3 Lipid Accumulation in response to WNT proteins in THP-1. Representative confocal fluorescent images of THP-1- derived macrophages. LipidTOX™ staining was used to evaluate the accumulation of neutral lipids within differentiated macrophages. The untreated (UT) controls were held for the duration of the *in vitro* experiments. Cells were treated with rWNT5A (100 ng/ml), rWNT3A (100 ng/ml), or in combination (both 100 ng/ml) as described in the Materials and Methods section. Cells were counterstained with 4′,6-diamidino-2-phenylindole (DAPI) and images are at 600X (scale bar = 50 μm). Quantification of lipid accumulation was represented as pixel intensity per cell as a measure of lipid droplet density and intensity. Data are representative of n = 2 experiments and are presented as bar graphs (mean ± SEM).
