# Supplementary figures and images for: Oxidized Low-Density Lipoprotein Induces WNT5A Signaling Activation in THP-1 Derived Macrophages and a Human Aortic Vascular Smooth Muscle Cell Line
Source: Front Cardiovasc Med. 2020 Nov 19;7:567837. doi: 10.3389/fcvm.2020.567837 (PMC7710548; doi:10.3389/fcvm.2020.567837)

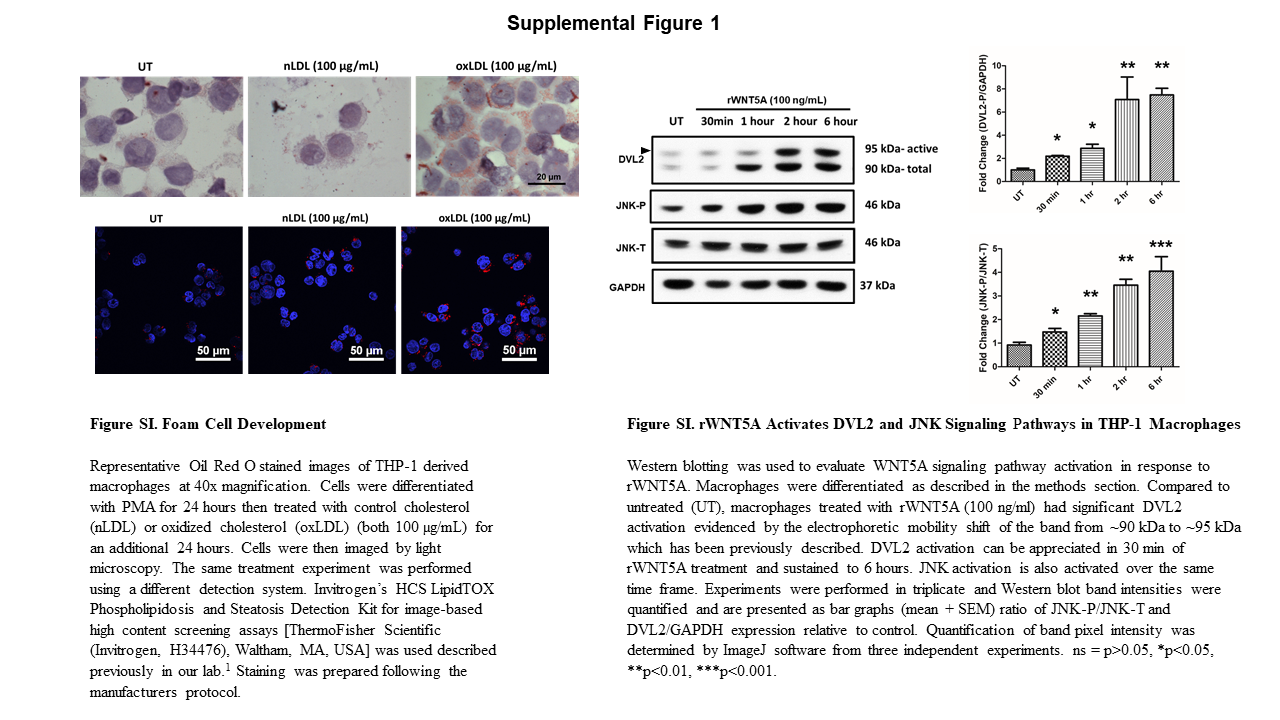

Supplement: Supplementary Figure 1 — Foam Cell Development and WNT5A Signaling. Representative Oil Red O-stained images of THP-1-derived macrophages. Cells were differentiated with PMA for 24 h and then treated with control cholesterol (nLDL) or oxidized cholesterol (oxLDL) (both 100 μg/mL) for an additional 24 h. Cells were then imaged by light microscopy. The same treatment experiment was performed using a different detection system. Invitrogen's HCS LipidTOX Phospholipidosis and Steatosis Detection Kit for image-based high content screening assays [ThermoFisher Scientific (Invitrogen, H34476), Waltham, MA, USA] were used previously in our lab. Staining was prepared following the manufacturer's protocol. Western blotting was used to evaluate the activation of WNT5A signaling pathway activation in response to rWNT5A. Macrophages were differentiated as described in the Materials and Methods section. Compared to untreated (UT), macrophages treated with rWNT5A (100 ng/ml) had significant DVL2 activation (black arrow), evidenced by the electrophoretic mobility shift of the band from ~90 to ~95 kDa which has been previously described (15). DVL2 activation can be appreciated in 30 min of rWNT5norA treatment and sustained to 6 h. JNK was also activated over the same time frame. Experiments were performed in triplicate and western blot band intensities were quantified and are presented as bar graphs (mean ± SEM) of the ratio of JNK-P/JNK-T and DVL2/GAPDH expression relative to control. Quantification of band pixel intensity was determined by ImageJ software from three independent experiments. ns = p > 0.05, *p < 0.05, **p < 0.01, ***p < 0.001. [file Image_1.TIF]

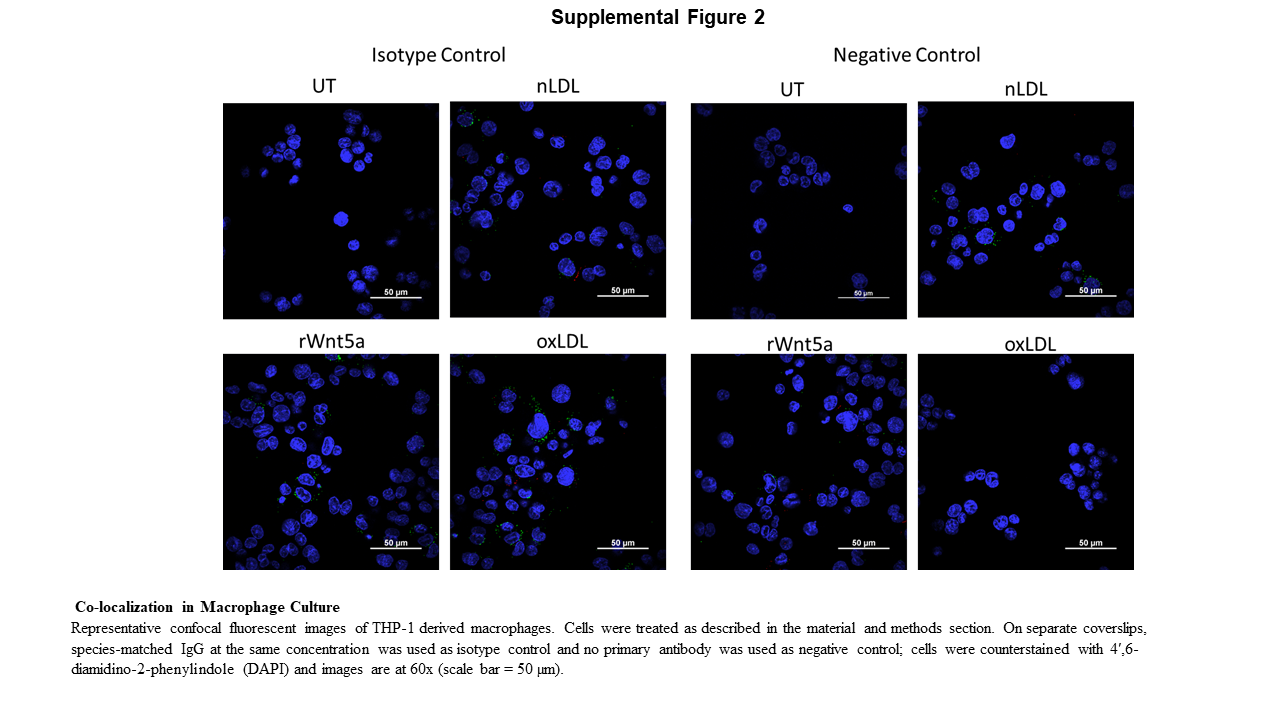

Supplement: Supplementary Figure 2 — Co-localization in Macrophage Culture. Representative confocal fluorescent images of THP-1-derived macrophages. Cells were treated as described in the Materials and Methods section. On separate coverslips, species-matched IgG at the same concentration was used as isotype control and no primary antibody was used as negative control; cells were counterstained with 4′,6-diamidino-2-phenylindole (DAPI) and images are at 600x (scale bar = 50 μm). [file Image_2.TIF]

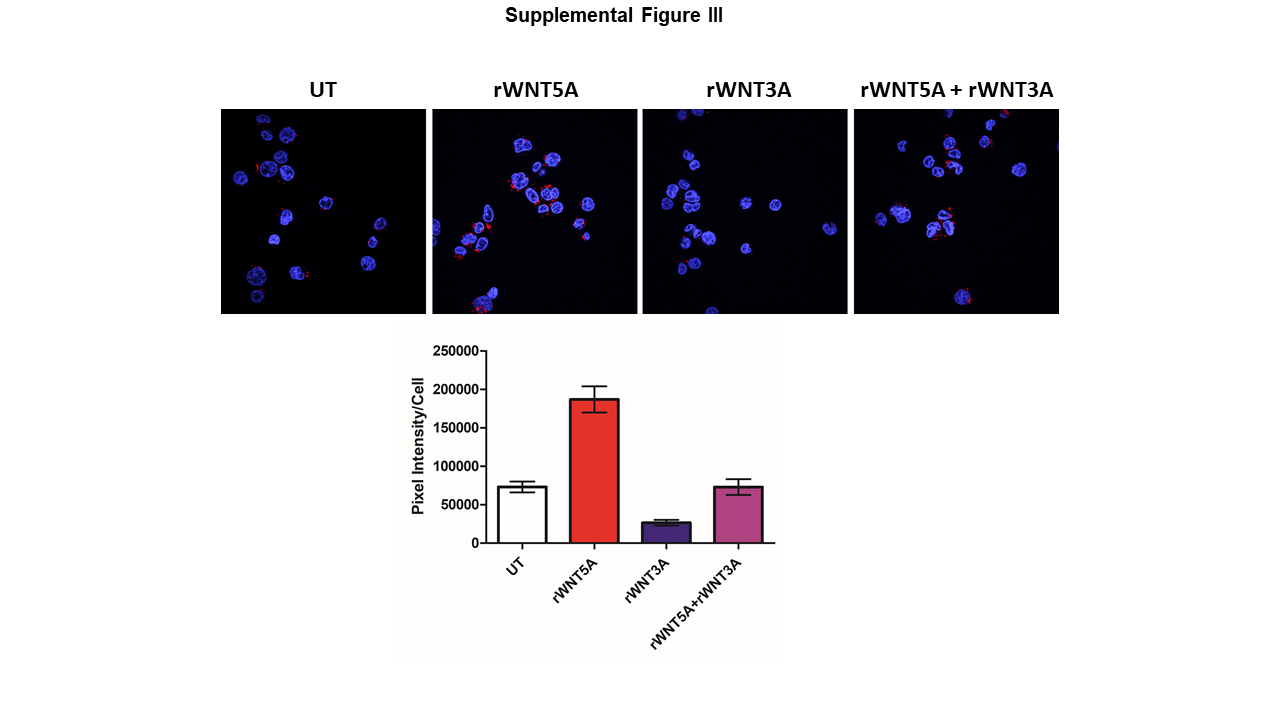

Supplement: Supplementary Figure 3 — Lipid Accumulation in response to WNT proteins in THP-1. Representative confocal fluorescent images of THP-1- derived macrophages. LipidTOX™ staining was used to evaluate the accumulation of neutral lipids within differentiated macrophages. The untreated (UT) controls were held for the duration of the in vitro experiments. Cells were treated with rWNT5A (100 ng/ml), rWNT3A (100 ng/ml), or in combination (both 100 ng/ml) as described in the Materials and Methods section. Cells were counterstained with 4′,6-diamidino-2-phenylindole (DAPI) and images are at 600X (scale bar = 50 μm). Quantification of lipid accumulation was represented as pixel intensity per cell as a measure of lipid droplet density and intensity. Data are representative of n = 2 experiments and are presented as bar graphs (mean ± SEM). [file Image_3.TIF]
